# PEAT: an intelligent and efficient paired-end sequencing adapter trimming algorithm

**DOI:** 10.1186/1471-2105-16-S1-S2

**Published:** 2015-01-21

**Authors:** Yun-Lung Li, Jui-Cheng Weng, Chiung-Chih Hsiao, Min-Te Chou, Chin-Wen Tseng, Jui-Hung Hung

**Affiliations:** 1 Institute of Bioinformatics and Systems Biology, National Chiao Tung University, Hsin-Chu, Taiwan; 2 Department of Biological Science and Technology, National Chiao Tung University, Hsin-Chu, Taiwan

## Abstract

**Background:**

In modern paired-end sequencing protocols short DNA fragments lead to adapter-appended reads. Current paired-end adapter removal approaches trim adapter by scanning the fragment of adapter on the 3' end of the reads, which are not competent in some applications.

**Results:**

Here, we propose a fast and highly accurate adapter-trimming algorithm, PEAT, designed specifically for paired-end sequencing. PEAT requires no *a priori *adaptor sequence, which is convenient for large-scale meta-analyses. We assessed the performance of PEAT with many adapter trimmers in both simulated and real life paired-end sequencing libraries. The importance of adapter trimming was exemplified by the influence of the downstream analyses on RNA-seq, ChIP-seq and MNase-seq. Several useful guidelines of applying adapter trimmers with aligners were suggested.

**Conclusions:**

PEAT can be easily included in the routine paired-end sequencing pipeline. The executable binaries and the standalone C++ source code package of PEAT are freely available online.

## Background

The paired-end sequencing technology, modified from the well-known single-end sequencing technology on next generation sequencing (NGS) platforms, plays increasingly important roles in genomics. By sequencing 5' ends of two strands of a DNA (or cDNA) fragment, it provides not just nucleic contents but also positional information of the fragment, therefore is a powerful resource to resolve the assembly in repetitive regions or structural variants. In addition, it is capable of obtaining interacting long-range DNA fragments[1], conveying information on both strands of the sequenced DNA, resolving exon junctions[2], and many other applications[3].

**Figure 1:**
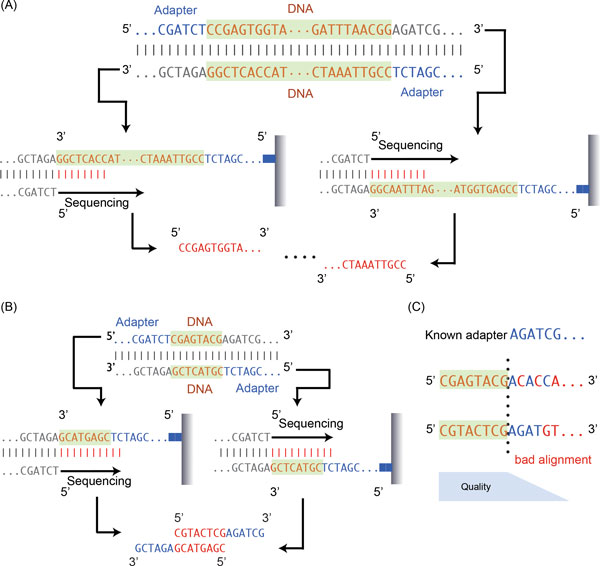
**Illustrations of paired-end sequencing**. (A) illustrates two strands of a double strands of DNA are both sequenced in the direction from 5' to 3' and ligated with paired-end adapters. In the situation that the double strands of DNA have a length longer than or equal to the machine specific sequencing length, no adapter corresponding sequences will be appended in the obtained paired-end sequenced reads. (B) illustrates the "read through" situation that the double strands of DNA have a length smaller than the machine specific sequencing length, so that parts of the adapter sequences are sequenced as well. (C) illustrates the error-prone strategy the existing adapter trimmers used for handling adapter-trimming operation.

A typical Illumina's paired-end sequencing technology is performed as follows: double strands of DNA fragments are both ligated with adapters and barcodes (when multiplexing), and then the 5' ends of the double strands are attached to the flow cell surface followed by many bridge amplification cycles to generate clusters for better nucleotide synthesis and fluorescence imaging. Both strands of each DNA fragment can serve as sequencing templates by regenerating the clusters, and consequently paired-end reads are produced (Figure 1A). According to the paired-end sequencing protocol of Illumina, users are allowed to choose the size-selected length of the DNA fragments (or inserts) from 200 to 500 base pairs (bp) with the sequencing quantity up to 200 millions of reads. Paired-end reads obtained are of a machine specific sequencing length, such as 36x2, 75x2, or 100x2 bp.

For DNA fragments that are at least as long as the pre-specified sequencing length (e.g., 100 bp), the sequencing process initiated from the 5' ends of both strands will be constrained and terminated before sequencing the adapters, so that only the 'real' DNA information is conveyed by the resultant paired-end reads (Figure 1A). However, in the case when the DNA fragments are shorter than 100 bp, the sequencer will 'read through' the real DNA into the adapters (Figure 1B). As a result, the paired-end reads generated will be appended with unwanted adapter sequences (namely, adapter contamination), and are likely dropped in the step of reference mapping.

To recover the "real" DNA part from the reads, existing algorithms rely on the correct alignment between adapter sequences against the 3' ends of reads. Such algorithms were originally designed for dealing with adapter contamination in single-end sequencing. They regard paired-end reads as two sets of single-end reads, and the adapter trimming is performed on each set independently. In practice, these single-end oriented adapter trimmers, such as FASTX[4], Cutadapt[5], ea-utils[6], TagCleaner[7], and Trim_Galore[8] (a wrapper of cutadapter for paired-end sequencing), do not give satisfactory results for paired-end sequencing. It is largely because current modern sequencers have a significantly higher sequencing error rate at the 3' ends of reads (Figure 1C). In other words, identifying the putative trimming positions by means of matching adapter sequence against read segments with poor sequencing qualities is highly error-prone. Some trimmers, such as AdapterRemoval[9], SeqPrep[10], and Trimmomatic[11], further filter positions found by the adapter sequencing scanning on each of the paired reads by examining the reversed complementarity between the untrimmed portions of the paired reads; however the accuracy still depends on the adapter sequence scanning on the error prone 3' ends. GATK ReadAdaptorTrimmer[12] looks for overlaps between forward and reverse reads in a pair to achieve high accuracy; however, it was designed specifically for streamlining with variant calling analysis pipeline in GATK[13], its input and output has to be in SAM/BAM format[14], which is cumbersome for other downstream analysis. The comparison of the performance of some of the trimmers has been investigated by several benchmark studies[15-17].

Here, we propose a highly efficient and accurate adapter-trimming algorithm and its implementation, PEAT (**P**aired-**E**nd **A**dapter **T**rimmer), designed specifically for paired-end sequencing. PEAT requires no adapter sequence input, which is particularly convenient when processing libraries using different adapters on a large scale. PEAT directly scans for the reverse complementarity between the good quality portions of the reads to avoid the loss of sensitivity during the filtering adopted by conventional approaches. We compared PEAT with many adapter-trimming tools. PEAT performed relatively well in simulated benchmarks and showed high scalability when applied to large real datasets. We applied PEAT to two public real datasets (101x2 paired-end sequencing libraries with 150 millions of total sequencing reads), multimillions of adapter-appended reads were successfully spotted, recovered, and mapped back to the reference. We further investigated the effects of PEAT to ten more real publicly available datasets of different sequencing applications such as ChIP-seq, MNase-seq, and RNA-seq. Comparisons between datasets processed with and without PEAT followed by the same typical downstream analyses revealed obvious pattern changes, which may deflect the biological notions toward the data. We suggest that more attentions need to be paid to adapter contamination in analyzing paired-end reads in all applications.

## Results and discussion

### An overview of the PEAT algorithm

In a typical Illumina paired-end sequencing protocol, a pair of adapter-appended reads emerges whenever a DNA fragment shorter than the pre-specified sequencing length get sequenced (Figure 1B). For a pair of reads sequenced from such a short fragment, the 5' ends of the reads, which correspond to the sequences of the real DNA fragment, have to be the reverse complement to each other (barcoding is ignored for simplification). The 3' ends, on the other hand, are corresponding to the adapter sequences, and should be equally long due to the fix pre-specified sequencing length.

**Figure 2:**
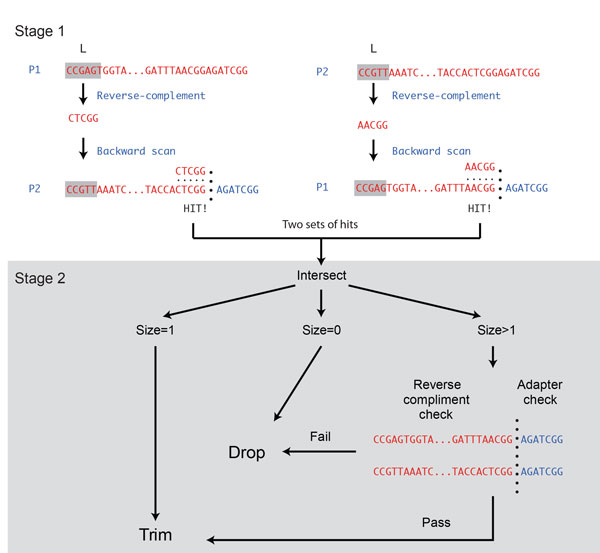
**Illustration of the algorithm that PEAT applies to handle paired-end adapter trimming operation**. The algorithm first conducts reverse-complemented string matching between the front parts of the paired-end sequenced reads of length *L*, which is pre-specified as a minimum possible DNA fragment length of the paired-end sequenced reads. The algorithm next verifies the trimming positions by identifying whether the accordingly determined front parts, i.e. the parts corresponding to the DNA fragments, are mutually reverse-complemented, and the rear parts, i.e. the parts corresponding to the adapter sequences, are substantially the same (optional). See *Methods *for details.

Based on the aforementioned observations, we propose a paired-end adapter-trimming (PEAT) algorithm with an efficient two-stage string matching strategy to detect the junctions between real DNA and adapters in paired-end libraries (Figure 2). The algorithm identifies multiple possible trimming positions by probing the reverse complementarity of the 5' end of the paired reads. To begin with, the reverse complement of a short 5' prefix (*L *nucleotides in length) of one read of the pair is used as a template to scan for mismatch-tolerant hits against another read of the pair. This procedure is performed twice by taking each read of a pair as the template iteratively and generates two possible sets of putative trimming sites. The intersection of the two sets is then used to check the reverse complementarity of the accordingly determined real DNA sequences (based on the given putative trimmed sites) of each read in a pair, and the parts corresponding to the adapter sequences, are substantially the same (in the case that forward strand and reverse strand adapters used in sequencing library preparation are totally different, the latter step can be skipped, see *Methods*). If none of the trimming position passes the check, PEAT reports that the pair is not appended with any adapter sequence.

By using 5' ends instead of 3' ends as both templates and targets for scanning, PEAT is able to determine the adapter trimming positions of the paired-end reads more accurately. PEAT is therefore more resistant to adapter contamination that is very short or having multiple adapter copies. Optionally, PEAT can also take the parts corresponding to error-prone adapter sequences as an auxiliary criterion for reexamination when the forward- and reverse-strand adapters share similar sequence (the E_3 _parameter, see *Methods*). PEAT also incorporates an open source single-end adapter trimming algorithm[18] to provide single-end adapter trimming functionality. PEAT supports multithreading for utilizing multiple computing nodes with data parallelism. Detailed algorithms and implementation can be found in *Methods *and the source code.

### Performance comparison

We evaluated the performance of PEAT, as well as other paired-end adapter trimmers, including AdapterRemoval, ea-utils, GATK ReadAdaptorTrimmer, SeqPrep, Trimmomatic, and Trim_Galore with six benchmark simulations and two real life datasets. The parameters of running these tools can be found in *Supplementary Methods *(Additional file 2). The benchmark simulations included datasets of three levels of quality: low-error-rate, middle-error-rate, and high-error-rate datasets, namely LED, MED and HED respectively, each of which had a million mock paired-end reads randomly sampled from a reference mouse genome (mm9) and half of the paired-end reads were selectively appended with the typical Illumina adapter sequences. Since typical Illumina libraries use the similar forward- and reverse-strand adapters, we also generated three companion dLED, dMED and dHED of which the prefix 'd' indicated that the reads were generated with the distinct forward- and reverse-strand adapters. In addition, we generated MED-o/dMED-o in which each paired-end read shared a 50-bp overlap (designated by the suffix -o) to demonstrate that our algorithm would not be fooled by extra reverse complementarity within reads. Mutations were introduced into reads according to a set of given sequencing quality scores (Figure 1, Additional file 1). The quality scores were sampled from a real dataset. Please see *Methods *for more details.

We ran PEAT and other trimmers on a Linux virtual machine and measure the execution time for trimming 1M of simulated reads of each of the eight datasets. All in all, with a linear-time algorithm (running time is proportional to the number of total bases, see *Methods*) effectively implemented with a lower-level computer language (i.e., C++), PEAT clearly achieved higher efficiency (FIG. S2, Additional file 1).

**Figure 3:**
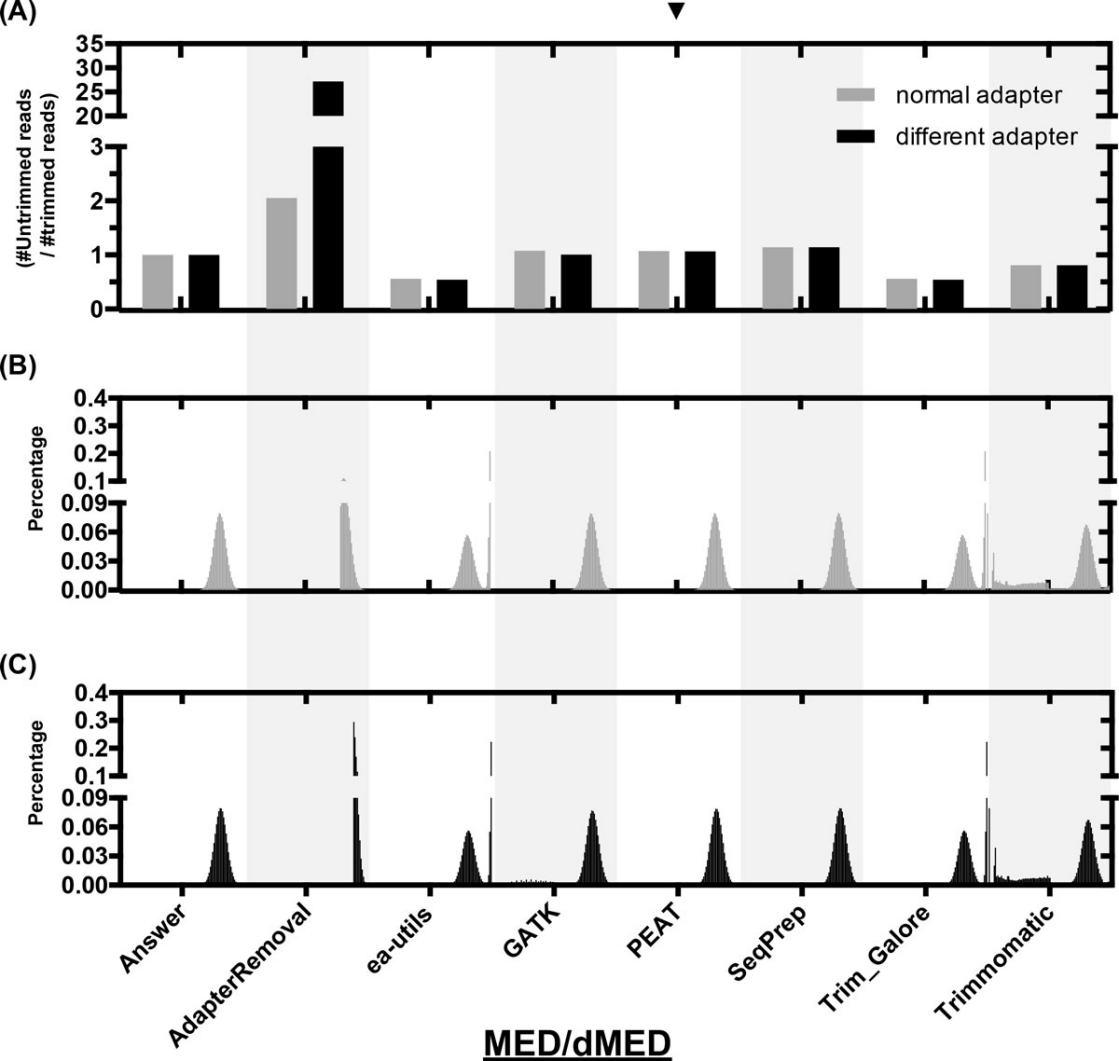
**Middle-error-rate (MED) datasets**. (A) shows the ratio of the read count of the untrimmed reads over that of the trimmed reads by all tested trimmers applied to the simulated MED/dMED datasets. (B, C) illustrates the length distributions of the trimmed reads processed by each of the tested adapter trimmers applied to simulated MED (B) and dMED (C) datasets. The distributions are depicted with the ratio of the amount of reads trimmed at certain length, ranging from 1 to 100 bp, over the total amount of trimmed reads. The ratios are magnified by 5 times with the range from 1 to 50 to visualize the presence of short fragments after trimming.

To evaluate the trimming performance, we calculated the sensitivity, specificity, accuracy, and Matthews correlation coefficient (MCC)[19] of all trimmers (FIG. S3, Additional file 1). Since some trimmers trim bases with low quality at 3' end and throw away reads that are too short after trimming by default, we used the options provided by the tools to make them behave similarly to the scenarios (see *Supplementary Methods*, Additional file 2). PEAT topped at two general metrics (accuracy and MCC) in most of the datasets. In general, the accuracy deteriorated when different adapters were used. AdapterRemoval seemed intolerant of different adapters in the paired-end libraries. Overlapping reads did not make apparent difference to the performance of all trimmers. We further investigated the ratio of trimmed reads over untrimmed reads (expected ratio: 1; Figure 3A and S4-6A, Additional file 1) and the length distribution of trimmed reads (Figure 3BC and S4-6BC, Additional file 1) obtained in eight datasets. The length distribution shows that some trimmers tended to trim a few bases at the 3' end (e.g., ea-utils and Trim_Galore), and some resulted in short fragments with a small chance (e.g., Trimmomatic and GATK ReadAdaptorTrimmer). Overall, the results of GATK ReadAdaptorTrimmer, PEAT, and SeqPrep showed only marginal differences to the gold standards.

We next applied PEAT and other trimmers to real life paired-end datasets (101x2) retrieved from GEO (GSM929772 and GSM929773, see *Methods*). In the comparison of trimming time with other tools, PEAT operated faster than AdapterRemoval, ea-utils, Trim_Galore, GATK ReadAdaptorTrimmer, SeqPrep and Trimmomatic by remarkable speedups of 20X, 2.4X, 2.7X, 9X, 13X, 7X respectively (see Table 1; no parallelism was applied in all the tests.). The result consists with that in the benchmark simulations, suggesting that PEAT is more efficient and also more scalable than most of the existing trimmers.

**Table 1:** Performance comparison of tested trimmers applied to two real life datasets.

	PEAT	AR^*^	EA^*^	TG^*^	GATK^*^	SeqPrep	TM^*^
**GSM929772 **(sequence depth: 152.8M)							

Time	1h 40m	36h 35m	3h 59m	4h 31m	14h 51m	22h 49m	12h 07m

# Trimmed	7,705,821	2,568,156	5,444,126	15,023,863	6,843,371	5,795,255	15,298,851

# Mappable	5,985,640	2,107,352	4,735,584	11,160,564	5,562,822	5,031,866	11,158,090

aTP	7,088,576	2,507,720	5,426,673	4,682,889	6,444,882	5,789,997	3,032,621

**GSM929773 **(sequence depth: 209.7M)							

Time	1h32m	33h 30m	3h 40m	3h 54m	13h 55m	20h 07m	10h 51m

# Trimmed	10,587,748	3,243,421	8,659,761	17,427,938	10,191,334	9,070,298	22,184,570

# Mappable	8,791,108	2,708,858	7,604,812	13,991,491	8,755,726	7,981,609	17,451,029

aTP	10,317,323	3,177,176	8,535,723	7,535,502	10,063,241	9,062,206	5,046,115

^*^Abbreviations: AR(AdapterRemovel); EA(ea-utils); TG(Trim_Galore); GATK (GATK ReadAdaptorTrimmer); TM(Trimmomatic)

PEAT identified 7.71M (5.04%) and 10.59M (5.05%) adapter-appended reads from GSM929772 and GSM929773 respectively (Table 1). The identified adapter-appended reads were further aligned back to the mm9 reference with end-to-end alignment by Bowtie2[20]. 5.99M and 8.79M reads were concordantly aligned one or more times to the genome, indicating that PEAT successfully had 77.68% and 83.03% of the identified adapter-appended reads of the two datasets properly processed and recovered. Since there was no gold standard, we simply took the number of reverse-complemented trimmed pairs to approximate true positives, and denoted it as aTP. PEAT gave the highest aTP among all tools. Comparing the insert length distribution of the original datasets to that processed by PEAT (FIG. S7 and S8, Additional file 1), we conclude that PEAT successfully recovered a significant amount of inserts shorter than 101bp without losing reads information conveyed in the original datasets.

For our tests, the results suggest that although some other trimmers also take advantage the reverse-complementarity to identify adapter contamination in different ways and also showed comparably performance, their algorithms do not scale very well and took much more time to complete the task. In addition, GATK ReadAdaptorTrimmer required extra 20+ hours of time just to transform between the formats for the two real life datasets (GSM929772 and GSM929772) in the tests. It could be a burden for users that do not intent to use the GATK framework.

### Applications in RNA-seq, ChIP-seq, and MNase-seq

In practice, the importance of adapter trimming is not fully recognized. It is not rare to assume that after size-selection--a common procedure to eliminate unwanted short fragments such as degradation products or rRNAs--DNA fragments having length shorter or greater than the size-selected length by a margin take only a very tiny part of the sequencing library. According to this assumption, DNA fragments with lengths outside the peak (either shorter or longer) are extremely rare, which makes some believe the lost of information carried by these minority is tolerable. We applied PEAT to two real life RNA-seq datasets GSM929772 and GSM929773 and provided evidence that this assumption might need to be adjusted (see FIG. S9, Additional file 1 and *Supplementary Methods*, Additional file 2).

**Figure 4:**
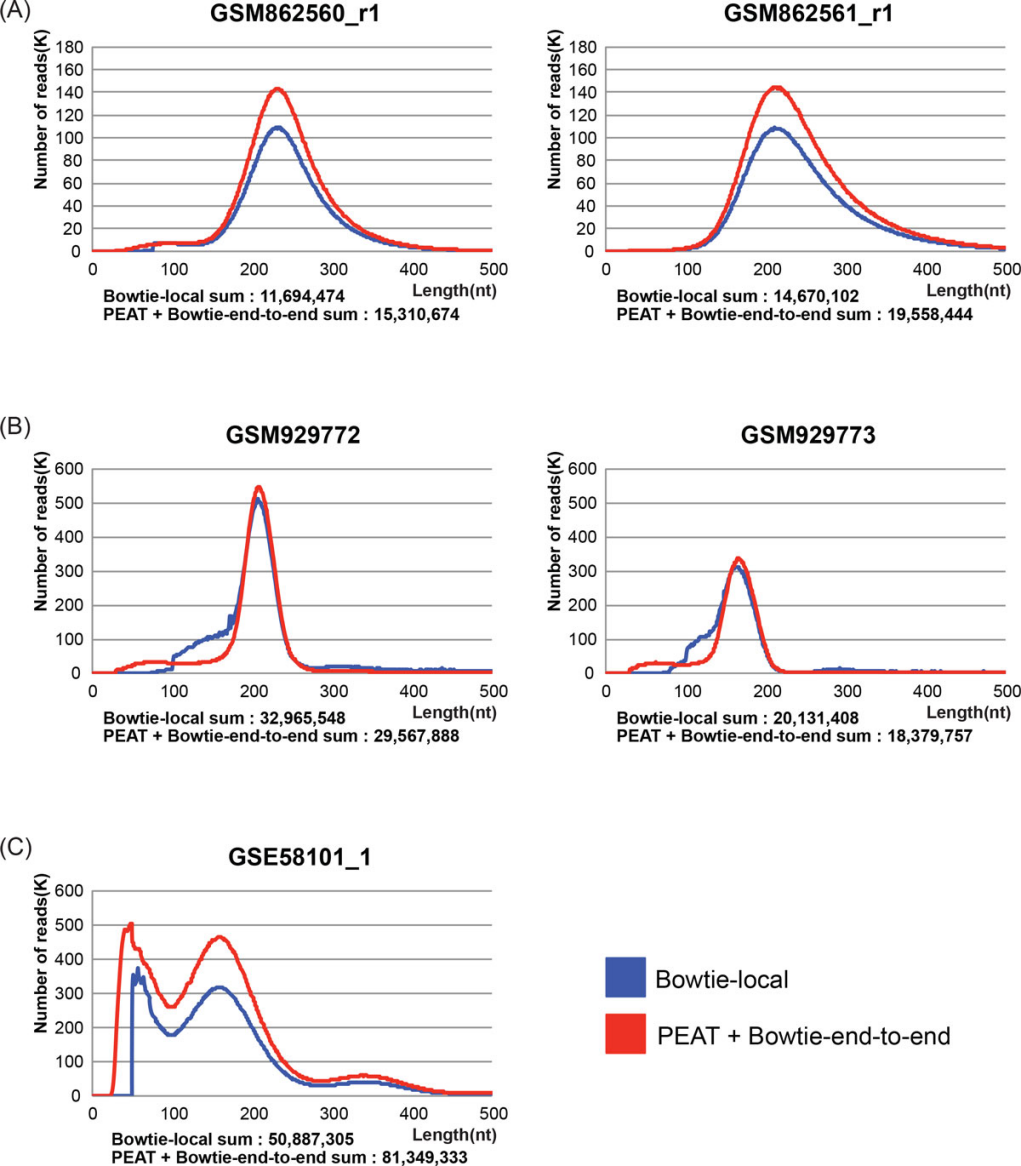
**The length distributions of the unique mapping reads from the selected sequencing libraries**. (A) ChIP-seq, (B) RNA-seq, and (C) MNase-seq datasets.

We further collected publicly available paired-end sequencing datasets for three typical NGS applications from GEO: RNA-seq (GSM929772-3, GSM1000574_1, GSM1000574_3), ChIP-seq (GSM862560_r1, GSM862561_r1, GSM862562_r1, GSM862563_r1) and MNase-seq (GSE58101_1-3), and we tested the influence on the downstream analyses when a competent adapter trimmer (i.e., PEAT) is included in the data processing. We used the local alignment option (--local) in Bowtie2 to relieve the problem of the non-mappability of the adapter-appended reads, which is a generally acceptable approach for the purpose[21] (see *Supplementary Methods*, Additional file 2). To eliminate possible miscalculation of insert length, we further excluded the multi-mapping reads and found significant changes in the length distribution (Unique mapping reads only: Figure 4 and S10, Additional file 1; with multi-mapping reads: FIG. S11 and S12, Additional file 1).

**Figure 5:**
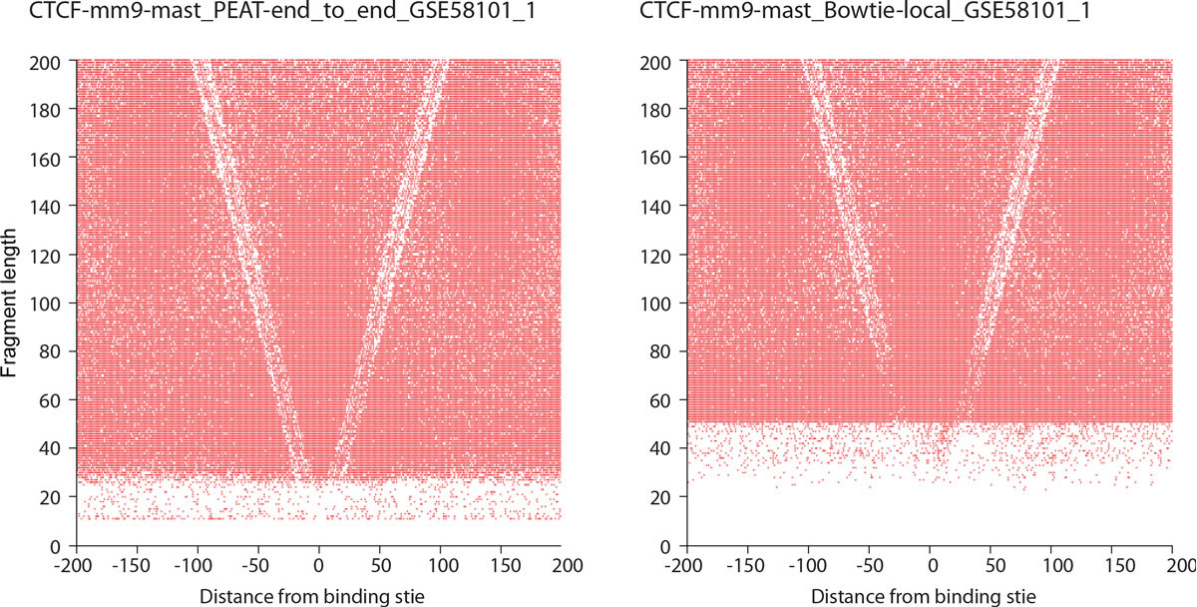
**The V-plots of the selected MNase-seq datasets**. The anchors were CTCF binding sites. The plot in the left panel was processed with PEAT and Bowtie2 end-to-end alignment option; the plot in the right panel was processed with Bowtie2 local alignment option.

In ChIP-seq datasets, we found that, without applying PEAT, unique mapping reads were reduced for about 40% (Figure 4A and S10A, Additional file 1), but after closer examination, the reduction were largely due to the use of local alignment, which led to fewer unique alignment. We didn't observe significant influence to the power of the downstream peak calling with adapter trimming (FIG. S13, Additional file 1. KS-test p-value: 0.051, 0.08, 0.068, and 0.052 for GSM862560_r1, GSM862561_r1, GSM862562_r1, GSM862563_r1 respectively); however, we would still suggest one should perform adapter trimming with Bowtie end-to-end alignment, since loosing 40% of unique mapping reads might be too costly just for getting rid of adapter contamination. In RNA-seq datasets, contrary to that in ChIP-seq and MNase-seq datasets (see below), about 10% more unique mapping reads were found without PEAT. We can see an additional surge of the blue curve (without PEAT) around 100-200 bp in Figure 4B and S10B, Additional file 1. We examined some of these alignments and found they were likely caused by the truncated junction reads (see FIG. S14, Additional file 1 for a typical case); therefore likely all the junction reads were reported shorter. However, using Bowtie2 with the end-to-end alignment option threw away all these junction reads. The best practice here should be using PEAT followed by a splice junction mapper like TopHat[22]. The short (<100 bp) and long (>100 bp) inserts found with PEAT were annotated to different populations (see *Supplementary Methods*, Additional file 2). Short inserts were enriched in snoRNAs and they were not found in the results without PEAT. In MNase-seq datasets, more than 30% of unique mapping reads were missed without PEAT (Figure 4C and 10C, Additional file 1). The absence of the short inserts affected the downstream V-plot analysis. The CTCF occupation profiles were less visible without adapter trimming, which may lead to wrong estimation of the size of the binding motifs (see *Methods*, Figure 5 and FIG. S15, Additional file 1). Along with these observations, we strongly suggest that a competent adapter trimming is crucial for the correct interpretation of the paired-end sequencing data.

## Conclusions

We herein propose the first paired-end specific adapter trimming algorithm and its implementation, namely PEAT. PEAT acknowledges the characteristics of current paired-end sequencing and takes advantage of the self reverse-complementarity nature of adapter-appended read pairs. It exploits a two-step process to search for reverse complement portions and accommodate high sequencing errors at the 3' ends. PEAT is written in C++ for more sophisticate control over memory and also equipped with multithreading for multiprocessor systems. Besides, with its reverse complementarity examining algorithm for 5' end, PEAT is able to operate without any adapter sequence input. This feature makes PEAT more suitable than other tools to handle large-scale sequencing data processes, in which different adapter sequences were used during library preparation.

Our simulation showed that single-end oriented trimmers that utilize post-processing to handle paired-end reads might be inappropriate. PEAT was able to determine the adapter trimming position of the paired-end sequenced reads with impressively high accuracy and efficacy. For the tests on real life datasets without gold standards, we used aTP to estimate the performance of trimmers and showed that PEAT was among the best choices. In addition, typical downstream analyses of ChIP-seq, RNA-seq, and MNase-seq were carried out and the results underlined the importance of a competent adapter trimmer.

PEAT currently is unable to handle barcode sequences in 5' ends, which will be improved in the future development. PEAT does not take into account of quality scores and indels. It is possible for PEAT to adopt a probabilistic model for scanning hits, although it is unpredictable if the model would help for better sensitivity or specificity, but it would likely reduce the speed. For sequencing platforms that introduce indels into reads (such as the 454 sequencers), PEAT doesn't perform well, but in the algorithmic aspect, it is not difficult to allow a fix number of gaps when scanning for hits without lose of efficacy. In addition, SOLiD reads in color space can be converted into nucleotide before processing by PEAT. We will support gapped alignment and color space alignment in the near future. The executable binaries and the standalone C++ source code package of PEAT are available at: [http://jhhung.github.io/PEAT].

## Methods

The rationale behind PEAT is to find the mutually reverse-complement portion between two paired reads. A naive implementation for an exhaustively search of all possible trimming sites of one read pair takes quadratic time. PEAT employs a two-stage algorithm to find the proper trimming positions in linear time without loss of specificity and sensitivity (Figure 1). The implementation details for the two stages of string matching are disclosed in the following paragraphs (see FIG. S16, Additional file 1).

### First stage of string matching

In the first stage of string matching, PEAT starts with an initial string matching length *L*, which can be set according to the minimum size of DNA fragments of interest. In current implementation, *L *is set as 30 by default. Let *R_1 _*and *R_2 _*denote the two reads of a pair. The first *L *characters of *R_1 _*and *R_2_*, denoted by *T_1 _*and *T_2 _*respectively, are selected. *T_1 _*is then reverse complemented and matched against *R_2 _*with a pre-defined mismatch-tolerance level (see below). All possible occurrences of reverse-complemented *T_1 _*in *R_2 _*(denoted by *G_1_*) signifies potential trimming sites where the 5' portion corresponding to the real DNA. PEAT performs the same matching procedure in the second round by aligning reverse-complemented *T_2 _*against *R_1_*, and obtaining another group of potential trimming sites *G_2_*. PEAT then performs set intersection on *G_1 _*and *G_2_*, to further extract highly reliable trimming sites (denoted by *I*) that are reported twice. If *I *is empty, PEAT reports *R_1 _*and *R_2 _*non-adapter-appended and works on the next read pair. If only one element is in *I*, PEAT simply reports it as the trimming site, and skips to the next read pair. While the intersection *I *has at least two elements, PEAT proceeds to the second stage of string matching (see below). The total required comparison in matching step is 2*L*N, where *N *is the length of a read. Since *L *is a small constant, the time complexity is therefore O(*N*).

### Second stage of string matching

The biggest element (*I_k_*) in *I *is taken, and the real DNA portions as well as adapter portions of *R_1 _*and *R_2 _*are retrieve according to *I_k_*. PEAT further examines the validity of the current trimming position (*I_k_*) by determining whether the two real DNA portions are reverse-complement to their counterparts by an error tolerant string matching. PEAT also performs an optional operation to test if the two resultant adapter parts are similar in sequence (this operation is turned on by default, and can be turned off by setting *E_3_*=1, see below) for libraries that use distinct 3' and 5' adapters). The validation of *I_k _*has O(*N*) time complexity. If the aforementioned criteria are met, PEAT accordingly regards the trimming position as valid, reports *I_k_*, and proceeds to the next read pair, otherwise PEAT removes *I_k _*from *I*, and repeats the second stage of string matching until the intersection group *I *becomes empty. Since *G_1 _*and *G_2 _*are constructed by scanning from the 5' end, they are sorted instinctually, *I *can be obtained in O(|*I*|). The size of *I *is small due to the small odds of having false positives (the sequencing errors more likely lead to false negatives than false positive), the cost can be amortized and the total time complexity of this stage is O(*N*).

### Error tolerant string matching

PEAT achieves error tolerance by setting a threshold on the ratio of perfect matches between two strings. The threshold corresponding to the first stage of string matching (*T_1 _*v.s *R_2 _*and *T_2 _*v.s. *R_1_*) is denoted by *E_1_*, which indicates that the maximum mismatches allowed is L*E_1_. *L *and E_1 _should be carefully chosen for adequate error tolerance. There are two additional thresholds in the second stage: the ratios for the reverse complementarity check (*E_2_*) of the DNA parts and that for the equality check (*E_3_*) of the adapter parts of *R_1 _*and *R_2_*. To ease the burden picking parameters, we fully test the combination of all *E *in different setups, and suggest to set *L*=30, *E_1_*=0.4, *E_2_*=0.6, and *E_3_*=0.4. All three thresholds can be adjusted by users for better performance regarding different library preparations. Users can set thresholds to one to turn off the corresponding steps.

### Simulation dataset generation

The system flow of the simulation dataset generation is illustrated in FIG. S17, Additional file 1. For generating adapter-appended read pairs, the length of each read was predetermined (101 nt), while the length of the adapter sequence (denoted as *A*) to be appended was sampled from a Gaussian distribution, *N*(*μ*=20, *σ*=5). Next, a reference genome, mm9, was used as a reference for generating a pair of adapter-appended reads. One randomly selected local sequence, with the length of the insert (i.e., 101-*A*), of the reference genome was obtained, and further reverse complemented to serve as its pairing counterpart. The well-conceived Illumina adapter sequences, 5'-AGATCGGAAGAGCGGTTCAGCAGGAATGCCGAGACCGATCTCGTATGCCGTCTTCTGCTTG (forward strand) and 5'-AGATCGGAAGAGCGTCGTGTAGGGAAAGAGTGTAGATCTCGGTGGTCGCCGTATCATT (reverse strand), were cropped and appended to the previously generated pair of reverse complemented sequence of reads to reach the predefined length. In the dHED, dMED, and dLED datasets, the adapter sequences used were 5'-CTAGAGTCAGTCCGGTTAATCCGGATCAGTCGTAGGAATCCAAAAGGTCCGTACGTACCTT (forward strand) and 5'-ATGGGCCCCTTTTAGTCAGTCAGTGGTTGGCCCTTTAAAATTTTCTCTTGAAGTCCCC (reverse strand). For adapter-free reads, on the other hand, two local sequences were randomly retrieved from the genome. In all cases, regions that have undetermined nucleotide were avoided. Each read was associated with quality scores based on the FastQ files acquired form a real dataset (GSM929772).

The encoded quality values were parsed and converted back to its numerical scales, each of which indicated the error probability of the corresponding nucleotide. In the generation of the MED, the error probabilities corresponding to each nucleotides were directly employed for mutation application, during the generation of the LED and HED, the error probabilities were scaled down or up by 10^0.5 ^times respectively. Mutations were introduced to each position according to the error probability. After that, reads generated were output in FastQ format with a sequentially generated read index number as the identifier and the corresponding quality values. In addition, we kept track of the identifiers together with its corresponding actual trimming sites, numbers of mutations in both DNA and adapter portions for the follow-up evaluation. Consequently, three sets of one millions of simulated paired-end sequencing reads (half adapter-appended), each of which was constructed from a reference genome segment, were selectively appended with a randomly determined length of adapter sequences, and randomly applied sequencing errors were introduced.

### Real life datasets

The RNA-seq datasets GSM929772, GSM929773, GSM1000574_1, GSM1000574_3 are all RNA-Seq libraries taken from Illumina Genome Analyzer provided by UCSC ENCODE data coordinating center. According to the documentation, polyA-selected RNAs were fragmented and converted into cDNA, and paired-end 2x101 bp reads were obtained from each end of a cDNA fragment. Paired-end libraries were further size-selected around 200 bp. The ChIP-seq datasets GSM862560_r1, GSM862561_r1, GSM862562_r1, GSM862563_r1 in was provided by GEO. The datasets were designed to determine the DNA binding sites of CTCF in mouse brain and contained paired 2x76 bp reads. Three MNase-seq datasets from GSE58101, which were called GSE58101_1, GSE58101_2 and GSE58101_3 in this paper.

### Downstream analyses

#### V plot analysis

The anchor CTCF binding sites were collected from Chen et al.[23]. We selected the top 1000 most significant peaks called by MACS 1.4 [24] with default setting from MNase-seq datasets and refined their borders by extending 20bp both up- and down-stream of the peak summits, which are the points with highest read coverage within peaks. We then generated V plots based on Henikoff et al.[25]. *Transcriptome annotation*: The annotations were gathered from the mouse genie informatics (MGI)[26]. *Peak calling*. The alignment results of ChIP-seq and corresponding input datasets were processed by MACS 1.4 with default setting.

## List of abbreviations

Next generation sequencing (NGS); Base pairs (bp); Low-error-rate dataset (LED); Middle-error-rate dataset (MED); and High-error-rate dataset (HED); Approximate true positives (aTP); Matthews correlation coefficient (MCC);

## Competing interests

The authors declare that they have no competing interests.

## Authors' contributions

JHH devised the algorithm and prepared the manuscript. YLL, JCW and JHH wrote the software. All authors contributed to the tests of the algorithm, presenting the results and preparing the figures.

## Supplementary Material

Additional file 1 **Supplementary figures and legends**. Click here for file

Additional file 2 **Supplementary methods**. Click here for file

## Declarations

This work was supported in part by grants from National Science Council (102-2311-B-009-001 and 103-2221-E-009-128-MY2), National Chiao Tung University and Ministry of Education (Junior Faculty Research Enhancement Project and Aiming for the Top University Program) of Taiwan to JHH.

This article has been published as part of *BMC Bioinformatics *Volume 16 Supplement 1, 2015: Selected articles from the Thirteenth Asia Pacific Bioinformatics Conference (APBC 2015): Bioinformatics. The full contents of the supplement are available online at http://www.biomedcentral.com/bmcbioinformatics/supplements/16/S1
