# Supplementary material for: PEAT: an intelligent and efficient paired-end sequencing adapter trimming algorithm
Source: BMC Bioinformatics. 2015 Jan 21;16(Suppl 1):S2. doi: 10.1186/1471-2105-16-S1-S2 (PMC4331701; doi:10.1186/1471-2105-16-S1-S2)
Supplement: Additional file 1 — Supplementary figures and legends. [file 1471-2105-16-S1-S2-S1.docx]

**Supplementary** **Figure Legends**


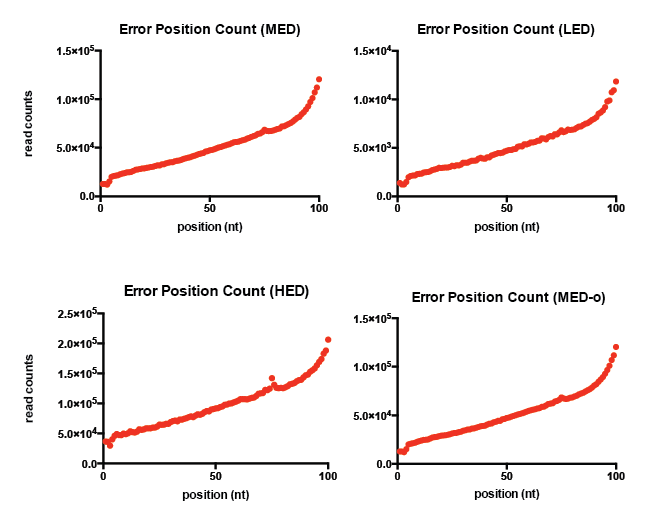


**Figure S1**. The amount of mutations introduced to each of the nucleotide position according to the quality scores for all simulated datasets.

**Figure S2**. Trim time comparison of eight simulated datasets.

**Figure S3**. The performance comparison for all trimmers with all eight simulated datasets.


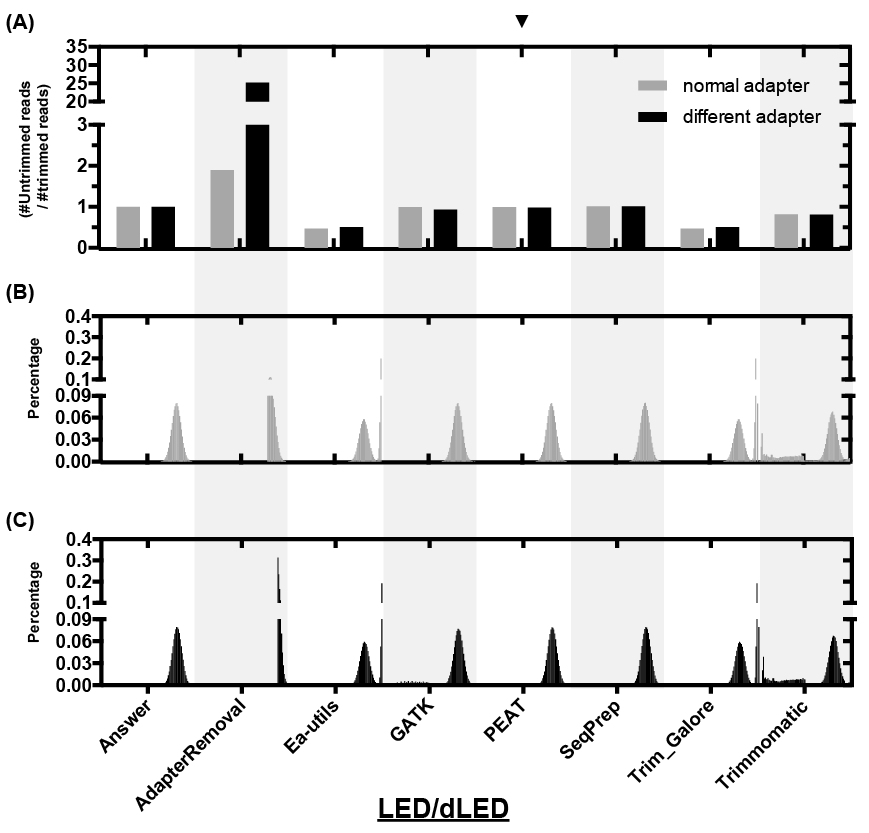


**Figure S4**. Low-error-rate (LED) datasets. (A) shows the ratio of the read count of the untrimmed reads over that of the trimmed reads by all tested trimmers applied to the simulated LED/dLED datasets. (B, C) illustrates the length distributions of the trimmed reads processed by each of the tested adapter trimmers applied to simulated LED (B) and dLED (C) datasets. The distributions are depicted with the ratio of the amount of reads trimmed at certain length, ranging from 1 to 100 bp, over the total amount of trimmed reads. The ratios are magnified by 5 times with the range from 1 to 50 to visualize the presence of short fragments after trimming.


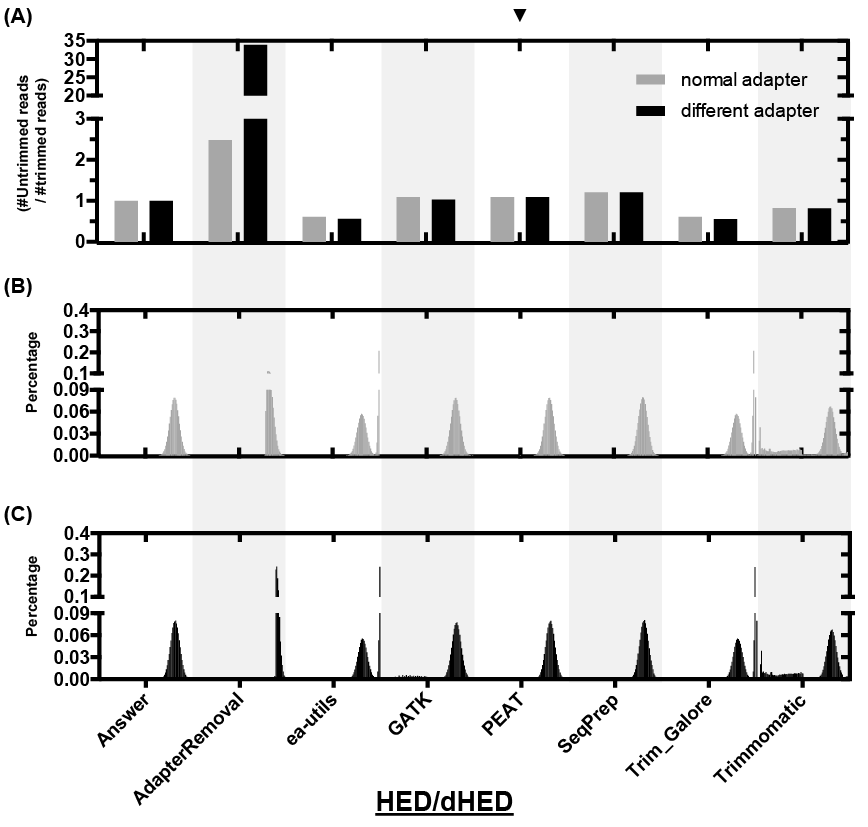


**Figure S5**. High-error-rate (HED) datasets. (A) shows the ratio of the read count of the untrimmed reads over that of the trimmed reads by all tested trimmers applied to the simulated HED/dHED datasets. (B, C) illustrates the length distributions of the trimmed reads processed by each of the tested adapter trimmers applied to simulated HED (B) and dHED (C) datasets. The distributions are depicted with the ratio of the amount of reads trimmed at certain length, ranging from 1 to 100 bp, over the total amount of trimmed reads. The ratios are magnified by 5 times with the range from 1 to 50 to visualize the presence of short fragments after trimming.

**Figure S6**. Mid-error-rate with overlapping reads (MED-o) datasets. (A) shows the ratio of the read count of the untrimmed reads over that of the trimmed reads by all tested trimmers applied to the simulated MED-o/dMED-o datasets. (B, C) illustrates the length distributions of the trimmed reads processed by each of the tested adapter trimmers applied to simulated MED-o (B) and dMED-o (C) datasets. The distributions are depicted with the ratio of the amount of reads trimmed at certain length, ranging from 1 to 100 bp, over the total amount of trimmed reads. The ratios are magnified by 5 times with the range from 1 to 50 to visualize the presence of short fragments after trimming.

**Figure S7**. The length distributions of the unique mapping reads of the real life dataset GSM929772. (A) illustrates the length distribution of DNA sequences conveyed in the real life dataset GSM929772, together with that treated by PEAT and other group two adapter trimmers, wherein the distributions of the raw reads and that of the PEAT processed reads are respectively illustrated. (B) illustrates the length distribution of DNA sequences conveyed in the real data set GSM92973 and that processed by PEAT and group two adapter trimmers we tested.

**Figure S8**. The length distributions of the unique mapping reads of two real life datasets. (A) illustrates the length distribution of DNA sequences conveyed in the real life dataset GSM929773, together with that treated by PEAT and other group two adapter trimmers, wherein the distributions of the raw reads and that of the PEAT processed reads are respectively illustrated. (B) illustrates the length distribution of DNA sequences conveyed in the real data set GSM92973 and that processed by PEAT and group two adapter trimmers we tested.

**Figure S9**. The length distributions of the unique mapping reads of two real life datasets. (A) illustrates the length distribution of DNA sequences conveyed in the real life dataset GSM929772, together with that treated by PEAT and other group two adapter trimmers, wherein the distributions of the raw reads and that of the PEAT processed reads are respectively illustrated. (B) illustrates the length distribution of DNA sequences conveyed in the real data set GSM92973 and that processed by PEAT and group two adapter trimmers we tested.

**Figure S10**. The length distributions of the unique mapping reads from the rest of the sequencing libraries. (A) ChIP-seq, (B) RNA-seq, and (C) MNase-seq datasets.

**Figure S11**. The length distributions of the multi-mapping reads from the selected sequencing libraries. (A) ChIP-seq, (B) RNA-seq, and (C) MNase-seq datasets.

**Figure S12**. The length distributions of the multi-mapping reads from the rest of the sequencing libraries. (A) ChIP-seq, (B) RNA-seq, and (C) MNase-seq datasets.

**Figure S13**. The distribution of the peak size from the peaks called by PEAT+Bowtie2 end-to-end alignment option (left) and by Bowtie2 local alignment option (right).

**Figure S14**. An illustration of a typical truncated alignment of paired-end reads. (A) The alignment of the P1 read; (B) The alignment of the P2 read. Only a small portion (first 22 bp) were aligned. The rest belongs to the next exon.

**Figure S15**. The V-plots of the selected MNase-seq datasets. The anchors were CTCF binding sites. The plots in the left panel were processed with PEAT and Bowtie2 end-to-end alignment option; the plots in the right panel were processed with Bowtie2 local alignment option.

**Figure S16**. The algorithm flow chart of PEAT.

**Figure S17**. The detail flow chart of the process of generating simulation dataset.
