# Supplementary material for: PEAT: an intelligent and efficient paired-end sequencing adapter trimming algorithm
Source: BMC Bioinformatics. 2015 Jan 21;16(Suppl 1):S2. doi: 10.1186/1471-2105-16-S1-S2 (PMC4331701; doi:10.1186/1471-2105-16-S1-S2)
Supplement: Additional file 2 — Supplementary methods. [file 1471-2105-16-S1-S2-S2.docx]

**Supplementary**

**PAET: an intelligent and efficient paired-end sequencing adapter trimming algorithm**

Yun-Lung Li^1^, Jui-Cheng Weng^1^, Chiung-Chih Hsiao^1^, Min-Te Chou^1^, Chin-Wen Tseng^1^ and Jui–Hung Hung^1,2,*^

*^1^Institute of Bioinformatics and Systems Biology, National Chiao Tung University, Hsin-Chu, Taiwan.*

*^2^Department of Biological Science and Technology, National Chiao Tung University, Hsin-Chu, Taiwan.*

^*^Address all correspondence to:

Jui-Hung Hung, Institute of Bioinformatics and Systems Biology, National Chiao Tung University, 75 Bo-Ai Street, Hsin-Chu, Taiwan 300, ROC

Phone: +886-3-5712121 ext 56991; E-mail: [jhhung@nctu.edu.tw](mailto:jhhung@nctu.edu.tw)

**Keywords**: paired-end sequencing, adapter trimming

**Supplementary methods**

**Comparison**. We evaluated the performance of PEAT, as well as other existing adapter trimmers, including AdapterRemoval, ea-utils, GATK ReadAdaptorTrimmer, SeqPrep, Trim_Galore and Trimmomatic with eight benchmark simulations and two real life datasets. All tools except PEAT and GATK ReadAdaptorTrimmer require *a priori* adapter sequences. For some tools, the length of adapters given affects the performance. To be fair, we gave the complete forward- and reverse-strand adapter sequences to all tools.

The gold standards were available for simulated data, so we can have the typical performance comparison. Only when both reads of an adapter-appended pair were trimmed at exactly the same position as expected was regarded as a true positive. False positives consisted of cases reporting different trimming sites on both reads of a pair or introducing any trimming site into an adapter-free pair. True negatives were cases reporting no trimming for an adapter-free pair as expected, and false negatives were those that report trimming in any one read of an adapter-appended pair.

**Options for applying trimmers**. All the currently existing trimmers provide some options to modify their adapter trimming configurations. We used their options to specify the appended adapter sequence as a whole or a segment of the following adapters of Illumina sequencing platform:

AGATCGGAAGAGCGGTTCAGCAGGAATGCCGAGACCGATCTCGTATGCCGTCTTCTGCTTG and

AGATCGGAAGAGCGTCGTGTAGGGAAAGAGTGTAGATCTCGGTGGTCGCCGTATCATT. Besides, for ea-utils, we ran fastq-mcf program with using -q 0 instead of the default parameter -q 10 to turn off quality trimming. We also applied -R to preserve ambiguous letters (Ns) from both ends of the reads to be consistent with other trimmers. For Trim_Galore, we used the --length 0 to preserve all trimming sequences to remain the consistency. For AdapterRemoval, similarly, we used --minlength 0 to preserve all trimming sequences and remain the consistency. For Trimmomatic, we used seed mismatches, palindrome clip threshold and simple clip threshold commands and set them in default parameters. In addition, Trimmomatic needed the adapter sequences to be input in the reverse complement form. When applied PEAT to simulated datasets, we used –l 50 option instead of the default –l 30 option, which increased the speed slightly. GATK ReadAdaptorTrimmer requires the input to be in SAM/BAM format; so extra format conversion was carried out with FastqToSam/SamToFastq commands. The complete commands we used for all the tools are listed in an additional supplementary file (tool_command.xlsx).

**Distribution of the length of size-selected inserts**. We used PEAT to show that the real life datasets GSM929772 and GSM929773, both of which are RNA-Seq datasets of mouse and are size-selected around 200 bp, do include a great amount of short DNA sequences (FIG. S7 and S8). PEAT successfully recovered a significant amount of short reads with length smaller than 100bp, which would have been discarded due to their incapability of mapping to the reference. We performed Kolmogorov-Smirnov tests to indicate that the insert length distributions of the PEAT processed real life datasets GSM929772 and GSM929773 and that of the best-fit Gaussian distributions (FIG. S9) are significantly different (p-values ≒ 0).
